# Decision support for risk prioritisation of environmental health hazards in a UK city

**DOI:** 10.1186/s12940-016-0099-y

**Published:** 2016-03-08

**Authors:** Mae Woods, Helen Crabbe, Rebecca Close, Mike Studden, Ai Milojevic, Giovanni Leonardi, Tony Fletcher, Zaid Chalabi

**Affiliations:** Centre for Radiation, Chemical and Environmental Hazards, Public Health England, Chilton, OX11 0RQ UK; Department of Social and Environmental Health Research, London School of Hygiene and Tropical Medicine, London, WC1H 9SH UK; Department of Cell and Developmental Biology, University College London, London, WC1E 6BT UK

## Abstract

**Background:**

There is increasing appreciation of the proportion of the health burden that is attributed to modifiable population exposure to environmental health hazards. To manage this avoidable burden in the United Kingdom (UK), government policies and interventions are implemented. In practice, this procedure is interdisciplinary in action and multi-dimensional in context. Here, we demonstrate how Multi Criteria Decision Analysis (MCDA) can be used as a decision support tool to facilitate priority setting for environmental public health interventions within local authorities. We combine modelling and expert elicitation to gather evidence on the impacts and ranking of interventions.

**Methods:**

To present the methodology, we consider a hypothetical scenario in a UK city. We use MCDA to evaluate and compare the impact of interventions to reduce the health burden associated with four environmental health hazards and rank them in terms of their overall performance across several criteria. For illustrative purposes, we focus on heavy goods vehicle controls to reduce outdoor air pollution, remediation to control levels of indoor radon, carbon monoxide and fitting alarms, and encouraging cycling to target the obesogenic environment. Regional data was included as model evidence to construct a ratings matrix for the city.

**Results:**

When MCDA is performed with uniform weights, the intervention of heavy goods vehicle controls to reduce outdoor air pollution is ranked the highest. Cycling and the obesogenic environment is ranked second.

**Conclusions:**

We argue that a MCDA based approach provides a framework to guide environmental public health decision makers. This is demonstrated through an online interactive MCDA tool. We conclude that MCDA is a transparent tool that can be used to compare the impact of alternative interventions on a set of pre-defined criteria. In our illustrative example, we ranked the best intervention across the equally weighted selected criteria out of the four alternatives. Further work is needed to test the tool with decision makers and stakeholders.

**Electronic supplementary material:**

The online version of this article (doi:10.1186/s12940-016-0099-y) contains supplementary material, which is available to authorized users.

## Background

In 2006, the World Health Organisation (WHO) estimated that 24 % of healthy life years (LYs) lost and 23 % of premature mortality were related to environmental factors [1]. A subset of these factors are environmental health hazards that have been linked to various acute and chronic diseases, such as carbon monoxide poisoning at a carboxyhemoglobin (CoHB) level of at least 10 % [2] and metabolic or cardiovascular disorders. Cardiovascular disorders contribute to 27 % of the death rate for all ages in Europe and are second only to neoplasms in the death rates of all ages across Europe [3]. In addition, environmental health hazards also contribute to a range of chronic diseases, such as asthma [4], autoimmune conditions including arthritis [5, 6], autoimmune thyroiditis [7], celiac disease [8] and multiple sclerosis [9]. Many of these health outcomes have shown relationships between exposure and disease. Examples include the molecular biology linking vitamin D with multiple sclerosis [10, 11] and the gene mutations associated with α particles from the short-lived radon-222 progeny [12, 13].

Government policies on existing interventions can help to manage the health burden caused by environmental health hazards by reducing exposure to such hazards. In this study we present a general decision support methodology for experts in public health. This methodology uses multi criteria decision analysis (MCDA), a method that can be applied to risk prioritisation for environmental public health (EPH) hazard interventions. In contrast to previous studies, we demonstrate how MCDA could be used in a local setting to combine both quantitative and qualitative evidence. The results obtained in this study are for illustrative purposes only, and are not necessarily reflective of the local situation in the city that we model. We focus on developing the methods that can be used to construct the MCDA evidence matrix with a hypothetical case study. Alternative policy evaluation methods exist, such as cost-benefit analysis (CBA) and cost-effectiveness analysis (CEA), and for a comparative analysis of CBA, CEA and MCDA, see [14]. CBA is based on the principal that all costs and benefits can be modelled with financial cost and uses the cost-benefit ratio to compare policies. CEA on the other hand uses the total cost per unit benefit in a criterion as the measure for comparative evaluation. Whilst both CBA and CEA methods provide information on the costs involved, it is difficult to quantify non-market impacts, such as environmental impact, morbidity and wellbeing. In contrast, MCDA is designed to handle multiple criteria in their different units and it could be argued that it is better at comparing policies across a wide range of impacts [14].

Previous studies in the literature have generated ranked lists of diseases and hazards based on epidemiological criteria, with the aim to inform policy makers on the set of hazards that should take priority. Current methods of prioritization generate quantitative information on the relative severity of each hazard with respect to one or two criteria, such as disability adjusted life years (DALYs) [15], cost of illness (COI) [15], quality adjusted life years (QALYs), quality of life (QoL) and relative risks [16]. For example, six microbial illnesses in New Zealand were ranked against DALYs and COI, where it was shown that Campylobacteriosis and sequelae ranked the highest for both [15]. In environmental health, Hollander et al., [16] have considered DALYs, life expectancy, QoL and the number of people affected for exposure to factors including particulate air pollution, radon, damp, environmental tobacco smoke and noise, placing particulate air pollution at the top of the ranking list.

Recently, upstream or distal causes of mortality and morbidity, such as road design factors have been considered as important in mitigating the environmental health burden for non-communicable diseases and road traffic injuries [17]. In contrast to the single or few proximal causes of an environmental health hazard, the set of upstream factors may be large and uncertain. The set of multiple confounding factors reside upstream of the immediate cause of premature death and suffering and can be regulated through a set of interventions. By ranking diseases with respect to one single health metric and proximal cause, multi-dimensionality that arises from upstream factors, implementation costs and intervention strategies are neglected. Studies that combine two criteria, such as the COI and DALYs address this issue in part, however there is no explicit impact of the intervention in the final ranking of environmental hazards. This final ranking will not be of practical value to make decisions on hazard management if there are no interventions available or in place. In summary, there is a need to develop models that quantify the performance of environmental health hazard interventions against criteria of public health concern. These models could act as transparent decision support tools for public health professionals. MCDA is one technique that can be designed to include proximal and upstream factors to support the complexities of multidimensional problems [18]. Transparency in MCDA is fundamental. Bots and Hulshof [19], argue that policy goals may fail if they are not transparent and define a five stage structure for participatory MCDA in the Netherlands. This transparent methodology can be applied in public health settings, where the management of one single environmental hazard is under deliberation. In Scotland [20], qualitative evidence was obtained for MCDA through consensus expert opinion for a set of flood management interventions. In addition, MCDA has been applied to developing air quality strategies and policy, reported by the UK Department for Environment, Food and Rural Affairs (DEFRA) [21]. A quantitative application of MCDA was applied in Belgium to rank chemical stressors and their associated health effects at different spatial scales [22]. There are detailed studies of MCDA outside the discipline of public health that demonstrate how quantitative and qualitative criteria can be evaluated. Caterino et al [23] demonstrate how MCDA can be applied on both types of criteria to determine stable structures in the presence of seismic activity, where they use the eigenvalue approach to convert qualitative variables to quantitative variables. Further analysis has been performed on this data set to investigate how uncertainty can be treated in MCDA using Dempster-Schafer theory [24]. In this study, we apply MCDA in public health. The focus of this work is to demonstrate how MCDA could be applied in a local setting to prioritise EPH interventions for a range of different environmental hazards against qualitative and quantitative criteria associated with the interventions. We have chosen four environmental hazards and these are presented in Table 1. In particular and in contrast to other applications of MCDA in environmental health policy, we demonstrate how MCDA can be implemented in a practical setting using a user-friendly web-based software (Annalisa © Maldaba Ltd 2009–2014, http://www.annalisa.org.uk/) which has been previously applied to support decision-making in patient centred health care [25]. Although MCDA is not a new methodology, implementation of MCDA in software such as Annalisa is yet to be applied in environmental public health hazard management.

**Table 1:** Environmental public health hazards, example associated interventions and health effects modelled for the case study

Hazard	Example interventions	Health effects modelled
Radon	Domestic buildings requiring remediation (e.g. retrofitting of active sumps, passive or active ventilation)	Lung-cancer mortality
Outdoor air pollution	Implementing local air quality management, emissions control (vehicular and industrial) and education	Chronic obstructive pulmonary disease
Indoor Carbon Monoxide	Fitting carbon monoxide alarms, servicing of gas appliances, ventilation, increasing awareness	Cardiovascular disease
Obesogenic environment	Encouraging walking and cycling, provision of cycle routes, encouraging the use of public transport, increased access to green spaces and fitness facilities, planning disincentives for fast food restaurants	Chronic obstructive pulmonary disease, all-cause mortality

The paper is divided into three main sections. The first section outlines the MCDA method. The second section describes the details of the case study to illustrate the use of the MCDA method and the third section discusses the findings.

## Methods

### Multi criteria decision analysis

MCDA is a quantitative method that can be applied to evaluate and compare alternative decision options (e.g. EPH interventions) in terms of their impacts on a set of criteria (e.g. mortality, morbidity, costs, environmental sustainability). The main steps of MCDA are (1) problem formulation where the decision options and the criteria are defined, (2) the construction of an evidence matrix which contains the impact of each option on each criterion, (3) weight elicitation which elicits (from decision makers) the relative importance of each criterion and (4) integration of the evidence matrix with the relative weights to provide an overall performance score of each option.

Mathematically, the overall score or value of each decision option is calculated using the following set of equations:

1 $$ {S}_i={\displaystyle \sum_{j=1}^n}{\omega}_j\times {\tilde{x}}_{ij} $$

2 $$ {\tilde{x}}_{ij}=\frac{x_{ij}}{y_j} $$

3 $$ {\displaystyle \sum_{j=1}^n}{\omega}_j=1 $$

Equation [1] gives the overall score *S*_*i*_ of option *i* on all the criteria, where *n* is the number of criteria. The coefficient *ω*_*j*_ is a normalised weight, which gives the relative importance of each criterion *j*. The weights should add up to unity (Eq. [3]). The variable ${\tilde{x}}_{ij}$ is an element of the evidence matrix, referred to as the rating of option *i* against the criterion *j* which is normalised by a suitably defined constant *y*_*j*_ such that, ${\tilde{x}}_{ij}=\frac{x_{ij}}{y_j}$. For each criterion *j* the set of ratings *x*_*ij*_ will have different units of measurement, consequently the ratings are usually standardised in scales of 0–1, 0–100 or 0–1000 [26]. The selection of the scaling constants is subjective. In this study for each criterion we used the sum of impacts across all options as the scaling constant.

### Weights

The weights *ω*_*j*_ are a key component of MCDA because they set the relative importance of each criterion. Every criterion *j*, is assigned a value of relative importance by the decision maker, or decision making team. Similarly to the ratings, weights are normalised between 0 and 1 and should add to unity across all criteria. This interactive allocation of the importance of each criterion is one example of the transparency of the MCDA approach.

### Ratings

Ratings ${\tilde{x}}_{ij}$ determine the impact of each object *i* on each criterion *j*. In contrast to the weights *ω*_*j*_, the ratings are calculated through mathematical modelling or expert elicitation. When all the ratings are evaluated, they form the evidence matrix. This analysis should be completed before the decision maker assigns weights to the criteria. In this study we present a general framework that could be employed to construct the evidence matrix by focusing on a hypothetical scenario. We will not be eliciting different weights on the criteria in this example, instead we will use a uniform weighting, where each of the weights will be assigned the same value, for illustrative purposes.

### Case study

**Fig. 1:**
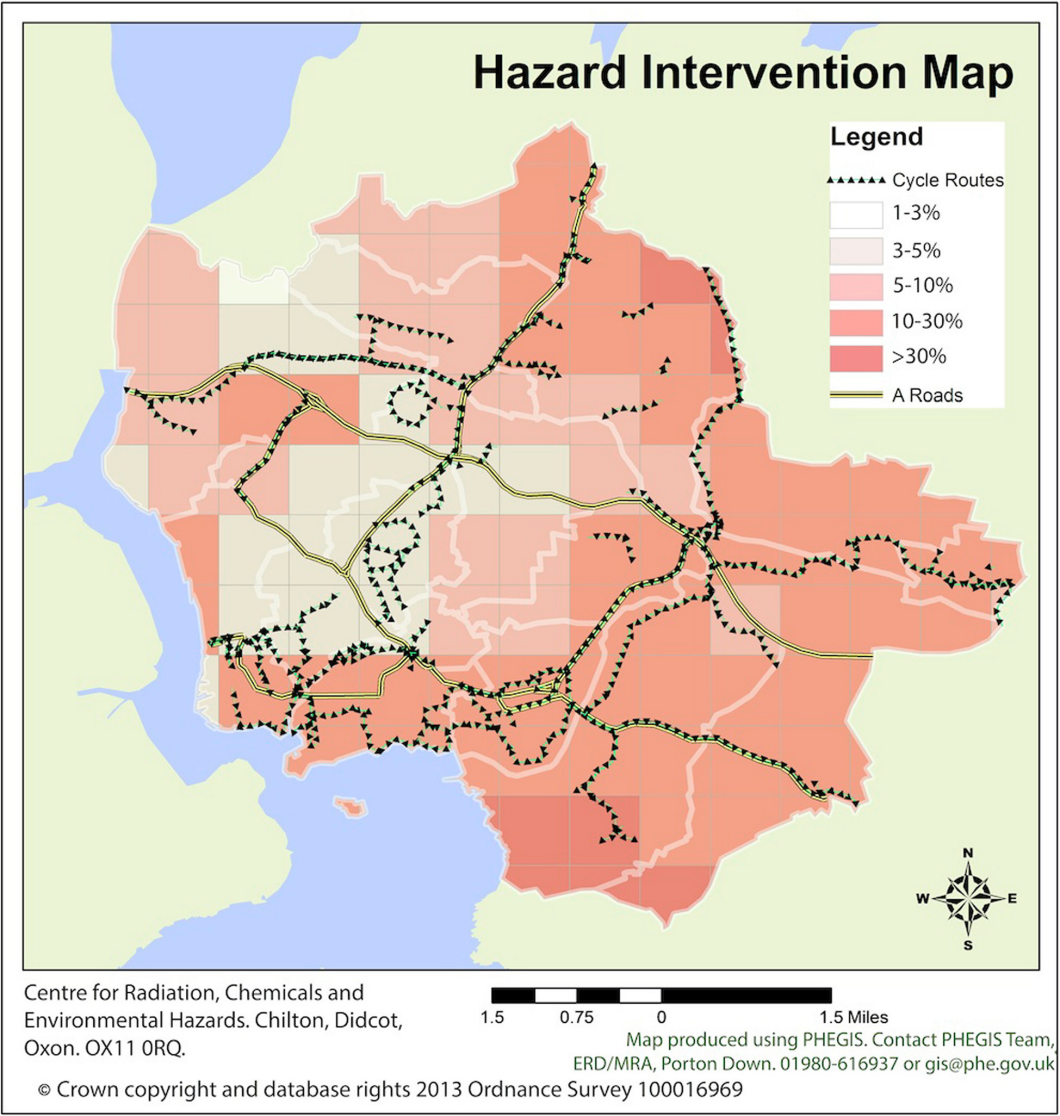
Example hazard and intervention map. Example city hazard and intervention map. Data were provided by Sustrans, GIS corporate datasets at PHE and the radon research group at PHE. © Crown copyright and database rights 2013 Ordnance Survey 100016969. Data that were used in the quantitative analysis include the A road junctions (thick bold lines), the local cycle routes, the national cycle network and the national cycle route networks (triangles), and the proportion of homes that exceed the action level for radon. We restricted all data included in the model calculations to the wards of the city (light gray lines). In the legend, boxes represent the percentage of homes predicted to be above the radon action level for the ranges 1–3 %, 3–5 %, 5–10 %, 10–30 % and >30 %

Here, we propose a method that can be used to design a MCDA tool to evaluate and compare interventions to reduce the health burden attributable to environmental hazards. The tool comprises three components: the set of criteria for comparing the interventions associated with the environmental hazards, models to determine the impacts of each intervention on each criterion (“ratings”) and the relative importance attached to each criterion (“weights”). The tool integrates the ratings and the weights to produce an overall score as a measure of how well the intervention performs across all the criteria for each intervention. The scores can be used to compare the performance of the interventions across all criteria and thus prioritise the interventions. Here, we focus on four environmental hazards and their associated interventions, just for example, with the aim of demonstrating the practical application of MCDA (see Table 1). This illustrative set of environmental hazards were chosen to be relevant to the ‘built environment’ and include both indoor and outdoor hazards. Decisions can be facilitated with MCDA by assessing the relative impacts of the interventions on a selection of criteria. In our scenario, we have chosen six criteria for the comparative evaluation of interventions: two quantitative criteria of mortality and morbidity and four qualitative criteria of ‘robust evidence’, ‘wellbeing’, ‘sustainability’ and ‘level of regulation’ (see Table 2). To obtain evidence for the qualitative criteria, we held a workshop to elicit expert opinion. Region specific data from an example city in the south west of England was used as evidence to assign parameters to the quantitative ratings. This data was collected from a variety of public sources, including Sustrans Cycle Network [27] and Public Health England (PHE) corporate datasets. Regional data sets were mapped in the graphical information system ArcGIS v10.0 [27] (see Fig. 1). The methods that were applied to obtain the ratings are presented in the following sections.

**Table 2:** Explanation of the quantitative and qualitative criteria used in the MCDA model for this case study

Quantitative criteria		
Mortality based on mortality models of relative risk from a change in exposure to the hazard following intervention. Morbidity based on hospital admission models of relative risk from a change in exposure to the hazard following intervention.		
Qualitative criteria		
Criteria	Application	Explanation
‘Robust Evidence’	Is there robust evidence for the risk?	What is the level of evidence on the risk, i.e. it is robust, plentiful, consistent, accepted by the scientific community
‘Wellbeing’	Impact on wellbeing	With the intervention in place, what impact does this have on wellbeing and happiness in particular
‘Sustainability of intervention’	Is the intervention sustainable?	Is the intervention sustainable in terms of economic, social, and environmental impacts? Does it require a lot of resources to keep in place and maintain? Are there social and environmental costs for its implementation and running?
‘Level of regulation’	How regulated is the intervention	Is the intervention subject to regulation? Is it enforceable in law? Are there penalties for failure? E.g. emissions tests.

### Quantitative criteria: mortality and morbidity

**Fig. 2:**
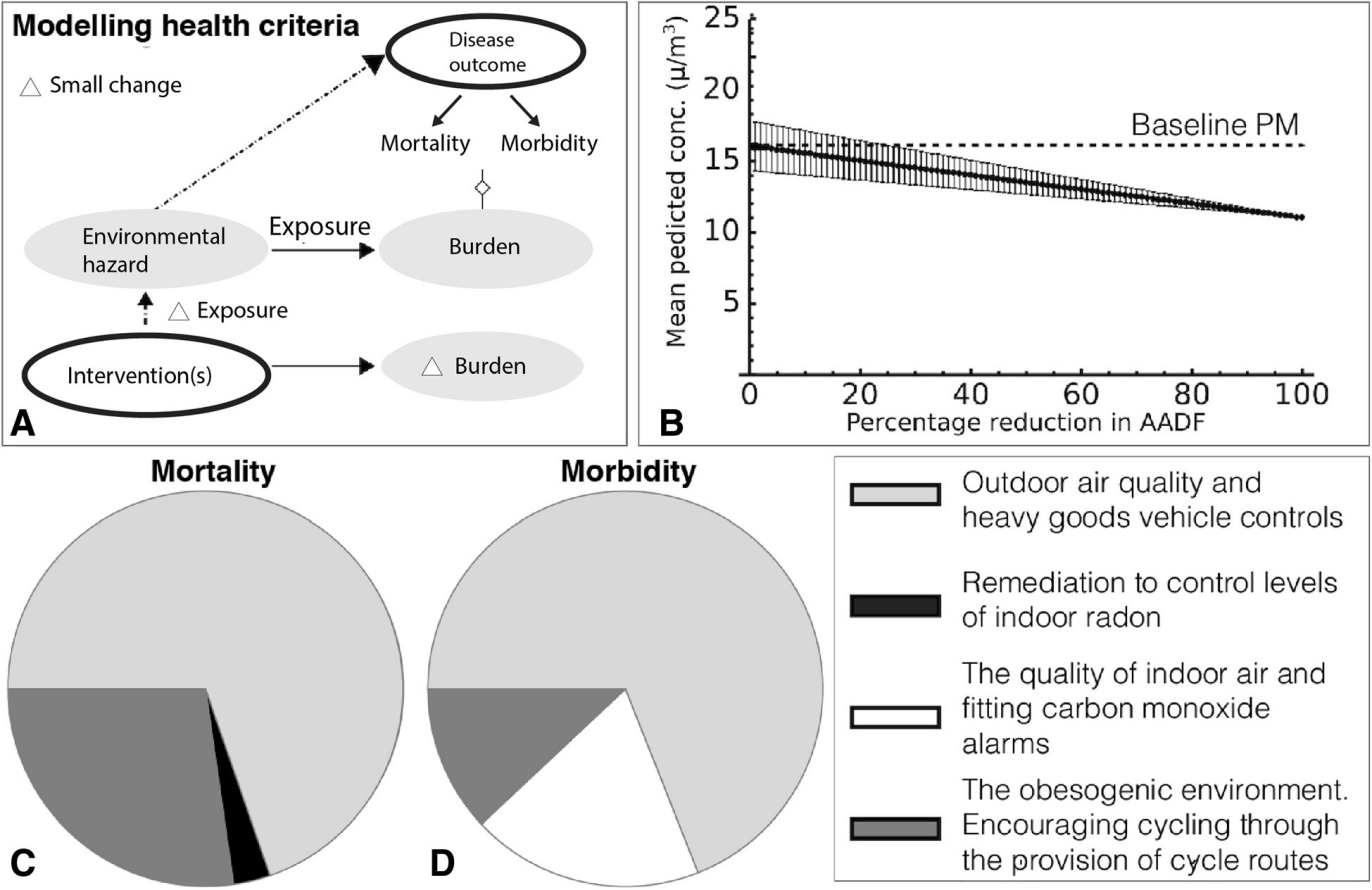
Mathematical modelling for ratings calculation. Mortality and morbidity impacts calculated for the set of hazards and corresponding interventions. **a**. Diagram depicting the modelling methodology applied to determine the impact of an intervention on the health burden associated with the corresponding environmental hazard. **b**. Decrease in PM_10_ as a result of percentage change in annual average daily flow (AADF) of HGVs calculated in CALINE4. The graph shows the mean PM concentration over seven estimates of the PM concentration within the city (solid line). Error bars represent one standard deviation from the mean. **c**. Pie chart showing the relative normalised ratings of the four hazards and interventions for the criteria mortality. **d**. Pie chart showing the relative normalised ratings of the four hazards and interventions for the criteria morbidity

For the criterion mortality, we applied models that evaluate the health impact of a change in exposure to the environmental hazard. These were used to calculate the intervention’s population preventable number of deaths, $$ \left({D}_{H_i}\right) $$, where the subscript *i* denotes the intervention (see Fig. 2a and Additional file 1). Excess relative risk ($$ ER{R}_{H_i} $$) for all-cause mortality, the baseline mortality rate for the city $$ \left({M}_{R_P}\right) $$ and the size of the affected population in the city at risk ($$ {N}_{P_i}=258,700, $$ obtained from the 2010 census [28]) were used to calculate a point (central) estimate of $$ {D}_{H_i} $$.

4 $$ {D}_{H_i}=\left({U}_i\times ER{R}_{H_i}\right)\times {M}_{R_P}\times {N}_{P_i} $$

In the above equation *U*_*i*_ is the intervention efficacy and in this study, we set *U*_*i*_ = 1 throughout, which assumes that intervention uptake is maximal. Equation [4] models the evidence that is associated with exposure to the environmental hazard and health effects of intervention. Here, it is used to calculate an approximate number of premature deaths prevented through hazard management. Regional values for ${N}_{P_i}$ and ${M}_{R_P}$ were obtained (see Additional file A1).

Similarly for the criterion morbidity, we use models of excess relative risk $\left(ER{R}_{H_i}\right)$ for cause specific hospital admissions, the population relevant disease prevalence (*d*_*R*_) and the size of the affected population in the city at risk (${N}_{P_i}$). These quantities are then multiplied to calculate a point (central) estimate of the morbidity impact *m*_*i*_.

5 $$ {m}_i=\left({U}_i\times ER{R}_{H_i}\right)\times {d}_R\times {N}_{P_i} $$

Equation [5] models the number of hospital admissions prevented by hazard management (interventions).

### Qualitative criteria

To obtain ratings for the qualitative criteria, we used an expert elicitation method by asking public health and academic experts to fill in a questionnaire distributed in a workshop. Originally, the following seven qualitative criteria were chosen: ‘Robust evidence for risk’, ‘impact on wellbeing’, ‘sustainability of intervention’, ‘level of regulation’, ‘acceptance of intervention by the public’, ‘acceptance of risk by the public’, and ‘prospect of intervention’. During the workshop, we only collected sufficient data from questionnaires on ratings from the first four criteria, and so the remaining criteria (i.e. ‘acceptance of intervention by the public’, ‘acceptance of risk by the public’, and ‘prospect of intervention’) were not considered in the MCDA analysis in this case.

The qualitative ratings were obtained by eliciting expert opinion on the performance of each intervention against the qualitative criteria on a scale from zero to one. Following the approach of Kenyon [20], questions were presented to academic experts in public health at a workshop on EPH and 15 min were allocated for the experts to provide distributions on the ratings for each intervention. To obtain the expert’s knowledge about the ratings, elicitation techniques were used to construct a probability distribution function for each of the ratings. Distributions were generated using a statistical method outlined in the software SHELF [29] and experts were asked to provide median, lower and upper quartiles for each rating. Each set of questionnaires addressed one intervention at a time and the questionnaires were designed to be accessible to decision makers with minimal prior knowledge of probability distributions.

The criterion ‘level of regulation’ was measured between “no regulations in place” and a scenario where “strict criteria and controls are maintained”. ‘Robust evidence of risk’ was graded between “no evidence available” and “significant high quality evidence available”. The final two criteria followed some existing definitions in the epidemiology literature. Sustainability was taken to concern the ‘environment, development, human needs and the capacity of the environment to cope with the consequences of economic systems’ [30]. Zero corresponded to a low sustainability and one corresponded to high sustainability of the intervention that can be defined by sustainability measures, i.e. the intervention is completely sustainable. For ‘wellbeing’, the questionnaire focused on the “happiness gained” as a result of intervention. We chose to focus on self-reported happiness, as it has been suggested that it is linked to the built environment [31].

## Results

### Example environmental hazards and interventions

#### Outdoor air quality and controlling the number of heavy goods vehicles

Particulate Matter (PM) has been associated with cardiopulmonary mortality [32] and morbidity [33] and the health burden can be reduced through compliance to air quality standards, vehicle emission controls and road traffic interventions [34]. To calculate the change in exposure to particulate matter less than 10 micrometres in diameter (PM_10_) from limiting the number of heavy goods vehicles (HGV), we focused on the percentage change in Annual Average Daily Flows (AADFs) for HGVs on the A road network in our example city (see Fig. 1). The AADFs provide the number of vehicles that will drive on a particular stretch of road on an average day of the year. Data and geometry of the AADF and A road network in the city were obtained from the open access traffic statistics published by the Department of Transport [35]. To obtain the rating for the impact of HGV controls on health, we calculated the reduction in the disease burden from changes in air pollution (PM_10_) that occur from a prospective percentage change in AADFs on the A road network in the city. The quoted relative risk for all-cause mortality for a 10 *μg*/*m*^3^ change in PM_10_ is 1.1006 in Europe [36], and so the relative risk coefficient is *β*_*PM*_ ≅ 0.01, (see Additional file 1). We calculated the point (central) estimate of the number of preventable deaths *D*_*PM*_ by reducing the flow of HGVs by 50 % on the A roads as an example air pollution control measure. This calculation required the predicted change in PM_10_, which we estimated using CALINE4 [37], one of many atmospheric emission dispersion software that are available. CALINE4 is an atmospheric dispersion model for line sources (road networks), and can be applied to a range of different geographical settings and where possible, we attempted to fit all parameters to existing data, using UK vehicle fleet averages and emission rates (see Additional file 2). This analysis predicted a change in PM_10_ of 2.5 *μg*/*m*^3^ (decrease per year), which could be substituted into Eq. [4] to obtain the number of deaths prevented *D*_*PM*_ ≅ 30 per year for the city. Next, we calculated the morbidity impact. The quoted relative risk of hospital admissions for chronic obstructive pulmonary disease (COPD) for a 10 *μg*/*m*^3^ change in PM_10_ is 1.38 [33]. The prevalence of COPD hospital admissions in the region of our city is ~0.2 % [38] and so *m*_*PM*_ ≅ 36 hospital admissions avoided per year. We only calculated morbidity impacts for COPD as an example of morbidity impacts.

### Remediation to control levels of indoor radon

Radon is a naturally occurring, radioactive gas that is produced from rocks and soils [39]. It is known that residential ingress of radon can increase the risk for lung cancer in the population, where the quoted relative risk of lung cancer incidence for a 100 *Bq*/*m*^3^ increase in measured radon is 1.16 [40]. Methods to reduce levels of indoor radon include built-in protection and remediation [41, 42]. There are various types of radon remediation available and in this example we chose the retrofitting of under floor ventilation in the form of active sumps for residential properties as an illustrative intervention [43]. It has been estimated that at most 2400 homes are at or above the action level in our city [44]. To obtain the regional population that would be affected by retrofitting this intervention, we assume the scenario that all houses that are at or above the action level could be remediated. This number of houses is multiplied by the average household size for the city of 2.3 persons per household [28], so that the estimated population that is exposed is around 5520 people. To estimate the reduction of indoor radon, we take the average level of radon for homes in the city, that warrant remediation, of 300 *Bq*/*m*^3^ which would be reduced by a reduction factor of 5.3 [43]. The reduction factor is the ratio of the initial radon concentration divided by the concentration after remediation. Using this ratio and rearranging the equation gives an approximate reduction of 243 *Bq*/*m*^3^. Previous studies on radon-related housing interventions have only considered health effects associated with lung cancer mortality [45]. To obtain the associated reduction in risk we used the age standardised crude mortality rate for lung cancer averaged between men and women. In 2011, this was 73.45 per 100,000 population in the city [46]. By substitution of the demographic values specific to our city into Eqs. [4] and [5], we obtained the number of preventable deaths *D*_*R*_ ≅ 1.2 per year. Relative to *D*_*PM*_, *D*_*R*_ is small, however this is expected because the number of people in the remediated homes initially exposed to indoor radon was approximated as 5520 (see supplementary Additional file 1).

### The quality of indoor air and fitting carbon monoxide alarms

Long term exposure to carbon monoxide (CO) has been associated with a range of adverse health effects, such as headache and exacerbation of COPD [47], self-reported neurological symptoms [48], angina in patients with ischemic heart disease, mild neurological effects, atherosclerosis, low birth weight [49] and congestive heart failure among the elderly [50, 51]. CO is a colourless, odourless gas and has a short half-life; symptoms are often similar to flu, which makes it difficult to identify and there may be many missed opportunities to intervene. Recently, a study on the prevalence of potential exposure to CO in Hackney Homes (inner London social housing) was conducted [52]. McCann et al. [52], estimated a CO alarm incidence rate of 4.64 incidence per 1000 households over a six month period, for CO levels above 50 ppm. Morris et al., [50] have calculated a relative risk of 1.22, associated with an increase of 10 ppm in CO for hospital admission due to cardiovascular disease (congestive heart failure). Typical background levels of CO in UK homes range from 0–48 ppm [53] and there are 108,000 households in our example city. Here, we assumed a worse case scenario of levels at around 50 ppm so that, at maximum efficiency, the intervention would reduce levels by 50 ppm. To calculate the mortality and morbidity ratings, we substituted the number of people exposed and the prevalence of heart failure of 0.7 % [54] into the Eq. [5]. This gave a result of *m*_*CO*_ ≅ 10 hospital admissions avoided. There are approximately 40 deaths per year due to acute carbon monoxide poisoning, although this figure is thought to be an underestimation of the true burden [55]. This value is small compared to the relative risk of hospital admission due to congestive heart failure. Thus *D*_*CO*_ ≅ 0.

### The obesogenic environment. Encouraging cycling through the provision of cycle routes and lanes

The obesogenic environment has been shown to have an effect on the prevalence of type 2 diabetes, dementia, ischaemic heart disease, cerebrovascular disease, breast cancer, colorectal cancer and depression [56, 57]. Interventions are being encouraged to help tackle obesity within the population and include promotion of cycling to work through the provision of cycle routes or lanes and cycle to work schemes with tax benefits. For this environmental hazard, the intervention is counterfactual. Our example city promotes cycling, by providing the following facilities; (1) cycle lanes (mandatory and advisory), (2) contra-flow cycle lanes, (3) contra-flow cycle streets, (4) cycle paths and (5) cycle tracks. These facilities are complemented by tax incentives for buying a cycle for commuting purposes through the Finance Act (1999) [58], which was introduced to promote healthier journeys to work and reduce environmental pollution. The quoted relative risk for all-cause mortality for an increase of 11 metabolic equivalent (MET) hours of physical activity per week is 0.81 [59, 60], where MET hours are measures of the energy cost of a physical activity per hour. In 2011, it was estimated that 3055 people in our example city travelled to work by bicycle and the average speed of a cyclist is 13.4 km/h [59]. To calculate the distance covered by the cycle routes in the city, we used the measure tool in ArcGIS. The distance covered by the national, local and National Cycle Network combined was 88,958 metres, which is less than the average distance covered for the average cyclist in a week of 93,730 metres. The average trip length for a UK cyclist per week was calculated by multiplying the average trip length (5150 metres [61]) by the number of trips per day (2.6) times seven (number of days in a week). To obtain the relative risk for this level of physical activity per week, we used the MET intensity for cycling of 8.5 [62] and the duration of hours cycled per week. By substitution of the values into Eq. [4], *D*_*o*_ ≅ 12 deaths avoided. To obtain the morbidity impact, we considered the number of hospital admissions associated with an increase in physical activity. For levels of moderate physical activity, the relative risk for COPD hospital admissions due to an increase in physical activity is 0.68 [63]. The prevalence of COPD hospital admissions in our region is ~0.2 % [38]. We approximated moderate physical activity with a value of 2 METs to calculate the exposure response coefficient. The values were substituted into Eq. [5] to calculate the morbidity impact: *m*_*o*_ ≅ 6 hospital admissions averted.

### Qualitative criteria: expert elicitation

**Fig. 3:**
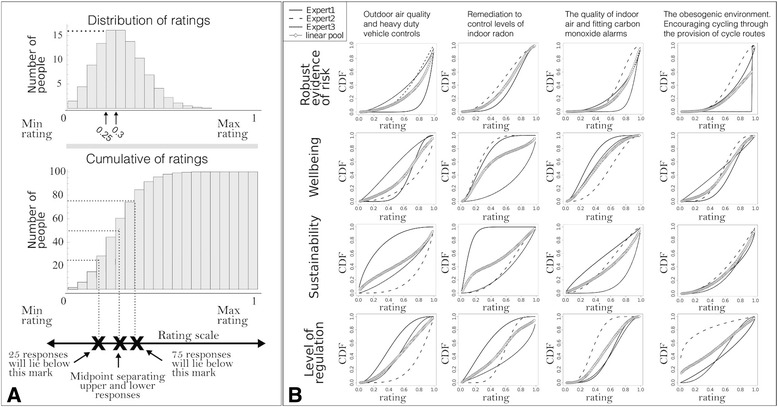
Elicited ratings for risk, wellbeing, sustainability and level of regulation. Expert-elicited evidence (ratings) of risk, wellbeing, sustainability and level of regulation calculated for the set of hazards and corresponding interventions. **a**. Figure showing the example presented to experts in EPH before completing the questionnaire to elicit summary variables for the ratings. **b**. Integration of the ratings for the qualitative criteria, using the software SHELF for each of the hazards and associated interventions. Plots show the individual cumulative distribution functions (CDF) and the overall linear pool

In each questionnaire, a short description of the object set and criterion was given and an example of the median, lower and upper quartiles were presented, (see Fig. 3a). To help the experts understand the illustrative criteria, they were given a descriptive rating scale that presented six equally spaced points on a continuous scale from zero to one, (see Fig. 3a). The results were imported into the statistical software SHELF [29] and three random expert opinions for each qualitative criterion were combined to obtain the consensus cumulative distribution function (CDF) (see Fig. 3b).

### Integrating the evidence

**Fig. 4:**
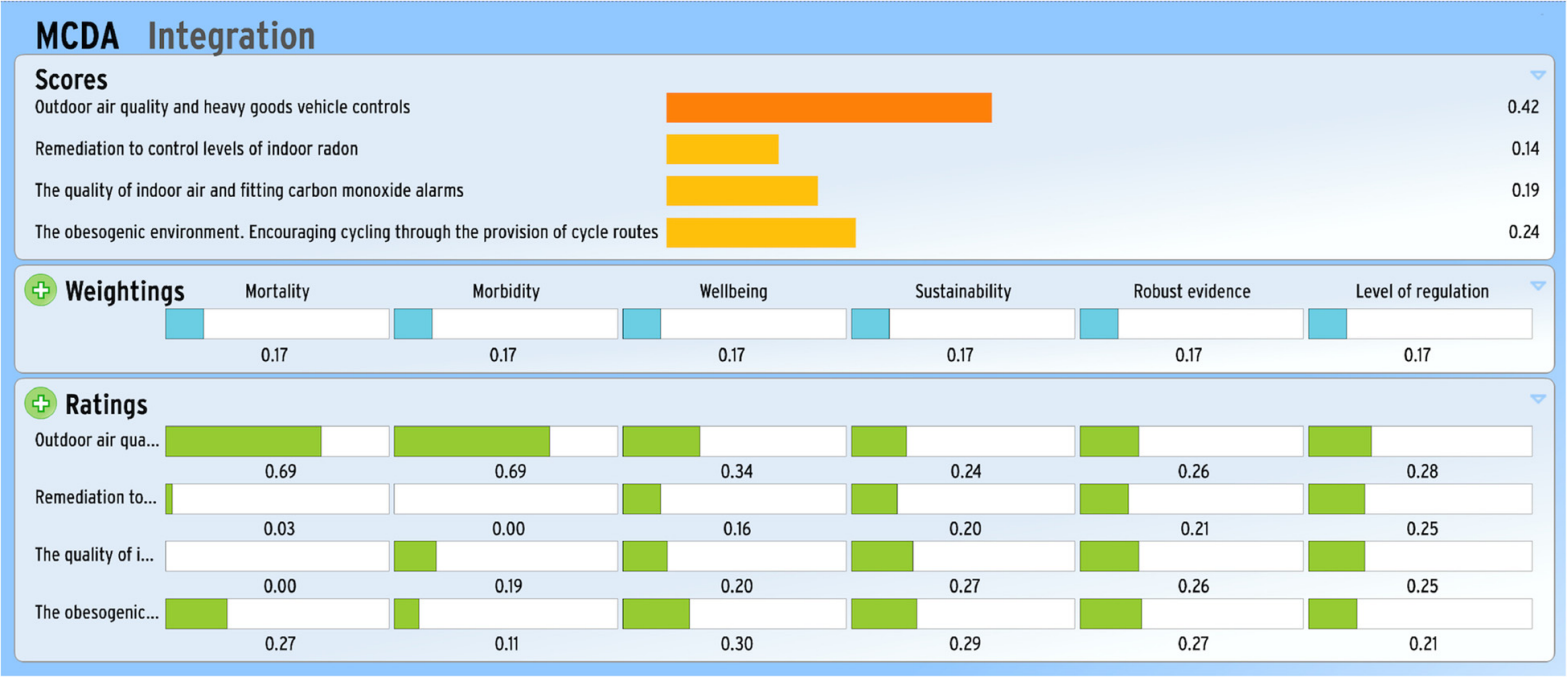
MCDA ranking of interventions and their associated environmental hazards. Extract (screen dump) from the model Annalisa. The MCDA tool was developed in Annalisa © Maldaba Ltd 2009-2014, (http://www.annalisa.org.uk/). Bottom panel shows values of the central point estimates of the normalised ratings that were calculated for the example city. Middle panel shows uniform weights, where in practice a stakeholder would be able to assign weights of importance. Top panel shows the integration of the ratings with the weights and priority of the interventions

The values of the evidence matrix, which feeds into the MCDA, are presented in Fig. 4. These values were obtained by normalising the ratings across the hazards and interventions. In this study, we apply uniform weights to the criteria and use an online MCDA decision support tool Annalisa [25] to determine the overall score of each intervention across all the criteria. The scores are then used to rank the interventions. In our illustrative example, HGV controls (to improve outdoor air quality) is ranked the highest, suggesting that this intervention has the highest positive impact across all criteria. Encouraging cycling was ranked the second highest, followed by the quality of indoor air and fitting carbon monoxide alarms. Remediation to control levels of indoor radon ranked the lowest. This was the outcome based on equal weightings of the criteria. Changing the values of the weights would have an effect on the final ranking order of the interventions. In a practical setting, sensitivity analysis can be performed to assess the robustness of the final ranks to changes in the weights, changes in exposure to the environmental hazard and intervention efficacy.

## Discussion

### Main findings

In this study we have demonstrated how MCDA could be applied in public health to evaluate, compare and rank interventions that mitigate the effect of environmental hazards on health across several criteria. The range and complexity of environmental health hazards to which the UK population is exposed is vast. Because of competing demands on resources, and a reduction in the public budget of governments, there is a need to prioritise the most significant environmental health hazards where interventions are likely to yield the greatest health benefit, whilst taking into account important factors such as the prospect of intervention and presence of statutory regulations. The MCDA methodology presented here is intended to provide a decision support framework for decision makers in a local authority who are responsible for distributing and allocating resources. Determining the best policy procedure is not intuitive, when dealing with multiple criteria. The decision problem is multidimensional in nature and as such, quantitative methods that combine the evidence based on the impact of interventions can be applied to rank interventions in terms of their performance across several criteria. The MCDA tool is designed to evaluate and compare interventions to reduce the health burden attributable to environmental hazards. Where local evidence has not been obtainable, we have used evidence from the literature to assign the relative risk of disease for local environmental hazards. We envisage that through a working partnership with experts who have practical experience of the current hazards and interventions required, that MCDA could help prioritise health hazards and their interventions and be applicable to provide quantitative predictions of the impact of policies that can reduce the health burden.

The use of MCDA to aid UK policy decision making is currently limited but has been applied to air quality [20] and flood defences [19]. Decision making usually relies on expert option as to how the proposal fits with national and local policy, the underpinning strength of evidence, the ease of its implementation, and the views of the public. The application of MCDA to support UK health policy is becoming more accessible with detailed studies on how to correctly apply MCDA, for example choosing among statins in primary healthcare prevention [64]. MCDA as a support tool in EPH fits with more strategic approaches including the use of ecological linkage frameworks that more explicitly incorporate the ‘distal’ determinants of health outcomes and related policy levers across a breadth of local settings. MCDA could be used within an ecosystems-enriched Drivers, Pressures, State, Exposure, Effects, Actions (eDPSEEA) conceptual model which integrates human health and environmental impact analysis. The model uses the concept of ecosystems services to emphasize human health and well-being values alongside the health of the environment [65]. Our application of MCDA has been shown to support the growing calls for ‘ecological public health’ as advocated by eDPSEEA.

### Study limitations

We presented a practical application of MCDA based on an example UK city. The scope of interventions considered here is minimal and was used only for illustrative purposes. There are many other interventions to manage the wide range of environmental hazards to which populations are exposed (e.g. waste management options). Similarly, the criteria considered is also minimal and does not reflect the range of criteria that would need to be considered for a practical application. For example, cost will vary considerably between each intervention chosen and often ultimately drives the decision. For a practical application of MCDA, experts in EPH could be approached for advice on which criteria to include in the MCDA model.

The purpose of our study was to demonstrate the potential benefit of using MCDA in supporting public environmental health decision-making. Thus, further data collection and analysis on both the quantitative and qualitative evidence would be required for a future in depth study and practical application. In particular, some excess relative risks refer to all-cause mortality and others specific-cause mortality. The evidence for the quantitative criteria is dependent on the availability of epidemiological data in the literature. Another study in this special issue presents scientific evidence for health affects due to indoor air pollutants [66]. In the study Asikainen et al. it is stated that current ventilation standards are specified by satisfaction with air quality. This approach to maintaining standards is limited because it does not elucidate health impacts due to long term exposure to indoor air pollution. By modelling three alternative exposure control scenarios, they found that indoor source control results in the largest health benefit. In a practical application of MCDA, it will be important for decision makers and experts in environmental health to be aware of the range of quantitative exposure impact models that are available, such as [66]. Where these models are available, the data should be incorporated into the MCDA evidence matrix.

The robustness of the final ranking of interventions could be investigated by performing MCDA under different scenarios and carrying out sensitivity analysis. For example, for outdoor air quality, a realistic intervention may not be able to reduce the flow of HGVs by 50 %. A 10 % reduction may be a more realistic target. We have used local data from a real city where we had data as an example to show how this could be applied to any population centre. In some calculations, local data was not available. Region specific data was obtained through local profiles of health and wellbeing within communities, such as from the Joint Strategic Needs Assessment, or County level health profiles [38, 46]. We assumed the road network only within our example city and not including neighbouring routes. Uncertainty could arise in the relative risk calculation, however with uniform weights, the final rank in our example is robust to perturbation in exposure and relative risk.

Limitations were also imposed on the analysis by the available data and assumptions made. For example in the radon assessment we have assumed that risk exists only for houses at or above the action level. A residual radon associated lung cancer risk also exists amongst those who live in houses below the action level, limiting the validity of our model of effect. Additionally, there are around 40 deaths per year due to carbon monoxide [66], however due to insufficient epidemiological evidence on relative risks, we have set the mortality rating for carbon monoxide to be zero. It is thought that the estimates of chronic carbon monoxide exposure are underestimated [42, 67], however, improved diagnosis and exposure estimates could help to gain a better understanding of mortality associated with carbon monoxide poisoning. For example, Davis and Cummings suggest the use of key diagnostic questions in clinical practice that could help identify CO related health outcomes [55].

In our pilot workshop, we produced questionnaires for seven criteria. These included: ‘acceptance of intervention by the public’, ‘acceptance of risk by the public’, and ‘prospect of intervention’ in addition to the four analysed in this study. To elicit a distribution of values from the data, we randomly chose three complete questionnaires. For each of these questionnaires, we required the upper and lower quartiles and the median values for each hazard and associated intervention. It was not possible to obtain these data for the criteria ‘acceptance of intervention by the public’, ‘acceptance of risk by the public’ and ‘prospect of intervention’. This was because a number of the participants had marked the box “don’t know” in the distribution. In a further study, it may be possible to survey the public on the acceptance of risk through questionnaires distributed to affected residents.

To integrate the evidence matrix with the weightings we applied uniform weights. Different weights across the criteria could be obtained by approaching experts in EPH, e.g. Directors of Public Health and environmental health practitioners in local authorities to elicit the weightings. When the final decision involves a group of stakeholders, different elicitation methods exist that can be used to integrate the individual weights, as outlined by Jia et al., [68]. For this example, we need to apply the model/tool to the real world, to test its application and obtain stakeholder feedback to add differential weights to the criteria that may affect the final ranking of the prioritised interventions.

## Conclusions

We have demonstrated that MCDA can be used in practice to support environmental health policy makers decide on the most appropriate interventions across a pre-defined set of criteria. The MCDA requires input on the impact of each intervention on each criterion (ratings) and on the relative importance of the criteria (weightings). The ratings can be obtained from models or elicited from experts. Weightings are normally elicited from decision makers. MCDA can be adopted as a practical, transparent tool to guide local authorities and environmental health policy makers. Further work is needed to test the tool with decision makers and stakeholders, where ease of use and compliance with the tool could be assessed and support provided where necessary. To apply MCDA in local authority settings for EPH, we suggest that decision makers can be supported in constructing the ratings by research scientists with expertise in epidemiology and exposure modelling. Once the ratings have been calculated, decision makers can then use decision support tools, such as Annalisa to perform the MCDA and obtain the ranking of intervention options.

## Additional files

Additional file 1:
**Calculations for quantitative criteria.** (PDF 8029 kb) Additional file 2:
**Parameters for atmospheric emission dispersion modelling.** (PDF 735 kb) Additional file 3:
**Peer review reports.** (PDF 118 kb)

## Abbreviations

AADF: annual average daily flow
CO: carbon monoxide
CoHB: carboxyhemoglobin
COI: cost of illness
COPD: chronic obstructive pulmonary disease
DALY: disability adjusted life year
DEFRA: Department for Environment, Food and Rural Affairs
EPH: Environmental Public Health
GIS: Graphical Information System
HGV: heavy goods vehicle
LSHTM: London School of Hygiene and Tropical Medicine
MCDA: multi criteria decision analysis
MET: metabolic equivalent
PHE: Public Health England
PM: particulate matter
QALY: quality adjusted life year
QoL: quality of life
SHELF: sheffield elicitation framework
UK: United Kingdom
WHO: World Health Organisation
