# Supplementary material for: Decision support for risk prioritisation of environmental health hazards in a UK city
Source: Environ Health. 2016 Mar 8;15(Suppl 1):29. doi: 10.1186/s12940-016-0099-y (PMC4895771; doi:10.1186/s12940-016-0099-y)
Supplement: Additional file 1: — Calculations for quantitative criteria. (PDF 8029 kb) [file 12940_2016_99_MOESM1_ESM.pdf]

Decision support for risk prioritisation of environmental health hazards in a UK city

Mae Woods[1,3§], Helen Crabbe[1], Rebecca Close[1], Mike Studden[1], Ai Milojevic[2], Giovanni Leonardi[1,2], Tony Fletcher[1,2] and Zaid Chalabi[2]

[1]Public Health England. Centre for Radiation, Chemical and Environmental Hazards, Chilton, Didcot, Oxon, OX11 0RQ

[2]London School of Hygiene and Tropical Medicine, Department of Social and Environmental Health Research, 15-17 Tavistock Place, London, WC1H 9SH

[3]University College London, Department of Cell and Developmental Biology, Medical Sciences Building, Gower Street, London, WC1E 6BT

[§]Corresponding author

Appendix A1.

Modelling mortality and morbidity risks for environmental hazards and interventions

For the criteria mortality, the MCDA ratings correspond to the number of environmental hazard attributable deaths that can be prevented by implementing an intervention. We assume that the health impact of each intervention is independent and we use the following equation to calculate the relative risk for each intervention.

$$RR_H = exp(\beta_H \Delta_e) \tag{1}$$

where RR_H is the relative risk associated with an increase or decrease in the chosen measure of exposure to the hazard (H). β_H is the exposure response coefficient for the hazard (H) for a unit change in exposure and Δe is the change in exposure. The value of RR_H for a specific change in exposure is obtained from the epidemiological literature. Based on this value, the risk coefficient can be calculated from equation (2)

$$\beta_H = \frac{\log(RR_H)}{\Delta e} \tag{2}$$

which is obtained by re-arranging equation (1). For any change in exposure Δ_{e_i} associated with intervention i, the relative risk is then given by,

$$RR_{H_i} = exp\left(\beta_H \Delta_{e_i}\right) \tag{3}$$

In equation (3), the relative risk RR_{H_i} corresponds to the change in exposure Δe_i to the hazard (H) induced by intervention (i). From equation (3), the excess health risk due to the intervention is given by:

$$ERR_{H_i} = RR_{H_i} - 1. \tag{4}$$

If $ERR_{H_i} > 0$ the excess relative risk due to the intervention is used to calculate a point (central) estimate of the number of preventable deaths,(D_{H_i}) and to represent the population, ERR_{H_i} is multiplied by the baseline mortality rate for the city M_{R_P} and the size of the population that benefits from the intervention, N_{P_i}.

$$D_{H_i} = ERR_{H_i} \times M_{R_P} \times N_{P_i}. \tag{5}$$

Equation (5) models the evidence that is associated with the environmental hazard and intervention and calculates an approximate number of deaths prevented through hazard management.

The estimated changes in exposure due to an intervention in all cases presented in this study were obtained from existing models in the literature. As such, these estimates will be uncertain. In addition, the parameter β_H used to calculate the relative risk was also derived from existing epidemiological models.

The calculations that were used to generate the central estimates of the preventable number of deaths from mitigation for each intervention are listed below.

Outdoor air quality and heavy goods vehicle controls

Mortality (per year)

$$\beta_{PM} = \log(1.1006)/10 \cong 0.01$$

$$RR_{PM} = e^{0.01 \times (-2.5)} \cong 0.98$$

$$ERR_{PM} = 0.98 - 1 \cong -0.02$$

$$d_R = \frac{578.3}{100000}, N_{PM} = 258700$$

$$D_{PM} = \left(\frac{578.3}{100000}\right) \times 258700 \times (-0.02) \cong -30$$

Morbidity (per year)

$$\beta_{PM} = \log(1.38)/10 \cong 0.03$$

$$RR_{PM} = e^{0.03 \times (-2.5)} \cong 0.93$$

$$ERR_{PM} = 0.976321 - 1 \cong -0.07$$

$$d_R = 0.002, N_{PM} = 258700$$

$$m_{PM} = (0.002) \times 258700 \times (-0.077) \cong -36$$

Remediation to control levels of indoor radon

Mortality (per year)

$$\beta_R = \log(1.16)\,/100$$

$$\beta_R = 0.0014842$$

where β_R is the exposure response coefficient taken from the literature [1].

$$RR_R = e^{0.001482\times(-243)} \cong 0.70$$

where RR_R is the relative risk for radon and $\Delta e_R = -243\text{Bq/m}^3$ is the expected reduction in radon.

$$ERR_R \cong 0.70 - 1 = -0.30$$

$$d_R = 73.45/100{,}000, N_R = 5520$$

$$D_R = (73.45/100{,}000)\times 5520\times(-0.3) \cong -1.2$$

D_R is the population preventable number of deaths, d_R is the age standardized mortality rate of lung cancer from 2011-2013, taken from [2] for our city per 100,000 population. We have taken the mean value for men and women between the years so that $d_R = 73.45/100000$.

The quality of indoor air and fitting carbon monoxide alarms

Morbidity (per year)

$$\beta_{CO} = \log(1.22)\,/10$$

$$\beta_{CO} \approx 0.02$$

$$RR_{CO} = e^{0.02\times(-50)} \approx 0.37$$

$$ERR_{CO} = 0.37 - 1 \approx -0.63$$

$$d_R = \frac{0.7}{100}, N_{CO} = 2305$$

$$D_{CO} = (0.007)\times 2305\times(-0.63) \approx -10$$

The obesogenic environment. Encouraging cycling through the provision of cycle routes

Mortality (per year)

$$\beta_{PA} = \log(0.81)\,/11$$

$$\beta_{PA} \cong -0.019$$

$$RR_{PA} = e^{-0.019\times(59.8)} \cong 0.32$$

$$ERR_{PA} = 0.31 - 1 \cong -0.68$$

$$d_R = \frac{578.3}{100000}, N_{PA} = 3055$$

$$D_{PA} = \left(\frac{578.3}{100000}\right)\times 3055 \times (-0.68) \cong -12$$

Morbidity (per year)

$$\beta_{PA} = \log(0.68)/6$$

$$\beta_{PA} \cong -0.06$$

$$RR_{PA} \cong e^{-0.06\times(59.8)} \approx 0.03$$

$$ERR_{PA} \cong -0.97$$

$$d_R = 0.002, N_{PA} = 3055$$

$$m_{PA} = (0.002)\times 3055 \times (-0.97) \cong -6$$

Table A1 - List of environmental parameters and ratings for quantitative criteria.

Hazard	Mortality statistic	Morbidity statistic
Radon	$\beta_R = 0.00148$	$\beta_R = 0$
	$RR_R = 0.7$	$RR_R = 0$
	$ERR_R = -0.3$	$ERR_R = 0$
	$d_R = 73.45/100{,}000$	$d_R = 0$
	$N_R = 5{,}520$	$N_R = 0$
	D_R= -1.2	D_R= 0
Outdoor air pollution	$\beta_{PM} = 0.01$	$\beta_{PM} = 0.03$
	$RR_{PM} = 0.98$	$RR_{PM} = 0.93$
	$ERR_{PM} = -0.02$	$ERR_{PM} = -0.07$
	$d_R = 578.3/100{,}000$	$d_R = 0.002$
	$N_{PM} = 258{,}700$	$N_{PM} = 258{,}700$
	D_{PM}=-30	D_{PM}=-36

Indoor Carbon Monoxide	$\beta_{CO} = 0$<br>$RR_{CO} = 0$<br>$ERR_{CO} = 0$<br>$d_R = 0$<br>$N_{CO} = 0$<br>D_{CO}= 0	$\beta_{CO} = 0.02$<br>$RR_{CO} = 0.37$<br>$ERR_{CO} = -0.63$<br>$d_R = 0.7/100$<br>$N_{CO} = 2{,}305$<br>D_{CO}=-10
Obesogenic environment	$\beta_{PA} = -0.019$<br>$RR_{PA} = 0.32$<br>$ERR_{PA} = -0.68$<br>$d_R = 578.3/100{,}000$<br>$N_{PA} = 3{,}055$<br>D_{PA}=-12	$\beta_{PA} = -0.06$<br>$RR_{PA} = 0.03$<br>$ERR_{PA} = -0.97$<br>$d_R = 0.002$<br>$N_{PA} = 3{,}055$<br>D_{PA}=-6

1. Darby S, Hill D, Auvinen A, Barros-Dios JM, Baysson H, Bochicchio F, Deo H, Falk R, Forastiere F, Hakama M, et al: **Radon in homes and risk of lung cancer: collaborative analysis of individual data from 13 European case-control studies.** *Bmj* 2005, **330:**223.
2. Maconachie M, Hoad S, Nelder R, Chant S: **Health and wellbeing profile. North, East and West Devon CCG and its three localities.** Public Health, Office of the Director of Public Health, Plymouth City Council; 2014.
