# Supplementary material for: Decision support for risk prioritisation of environmental health hazards in a UK city
Source: Environ Health. 2016 Mar 8;15(Suppl 1):29. doi: 10.1186/s12940-016-0099-y (PMC4895771; doi:10.1186/s12940-016-0099-y)
Supplement: Additional file 2: — Parameters for atmospheric emission dispersion modelling. (PDF 735 kb) [file 12940_2016_99_MOESM2_ESM.pdf]

Decision support for risk prioritisation of environmental health hazards in a UK city

Mae Woods[1,3§], Helen Crabbe[1], Rebecca Close[1], Mike Studden[1], Ai Milojevic[2], Giovanni Leonardi[1,2], Tony Fletcher[1,2] and Zaid Chalabi[2]

[1]Public Health England. Centre for Radiation, Chemical and Environmental Hazards, Chilton, Didcot, Oxon, OX11 0RQ

[2]London School of Hygiene and Tropical Medicine, Department of Social and Environmental Health Research, 15-17 Tavistock Place, London, WC1H 9SH

[3]University College London, Department of Cell and Developmental Biology, Medical Sciences Building, Gower Street, London, WC1E 6BT

[§]Corresponding author

Additional file 2. Parameters for Atmospheric Emission Dispersion Modelling

To run the CALINE4 software, regional parameters must be defined. The regional parameters specify the geometry and physical surrounding of the road network. Parameters consist of the concentration of the pollutant that is to be predicted, its molecular weight, settling velocity, deposition velocity and the surface roughness of the area concerned and ambient PM concentration (background levels, from other sources). Meteorological parameters can be set and include the wind speed, direction, atmospheric stability, mixing height, and temperature. Geometry inputs specify the local road network, where each link between the road nodes can be assigned a type. Finally, the link activity includes the traffic volume in units of vehicles per hour. The list of inputs for the wards of our city are listed in table A1. Some of the values are set at the default level and others are specific to the case study. This application of CALINE4 is illustrative and to apply the tool in a practical example experts in EPH could be approached for region specific parameter values. In a practical application, the results of the atmospheric dispersion model should be compared for different parameter values and sensitivity analysis performed. Parameter values that could be varied include the atmospheric stability class, which ranges from 1-7.

Table A1- Input parameters for the model run in CALINE4

**Input**	**Value**	**Reference**
Molecular weight	NA	Not required for particles

		(PM_{10})
Settling and deposition velocity	$0.1 cm/s$	A value that is optional for PM, taken from the Caline4 manual [1]
Aerodynamic roughness coefficient	400	Corresponding value specified for a city centre, taken from the Caline4 manual [1]
Run type	Worst case wind angle	Recommended in Caline4 documentation, taken from the Caline4 manual [1]
Altitude above sea level	$50m$ (above mean sea level)	Height ASL for our city from OS maps/GIS [2]
Number of links	20	Maximum input for Caline4, chosen to best approximate the road network. Specified in the Caline4 manual [1]
Number of receptors	7	to represent urban background receptors within wards of our city Specified in the Caline4 manual [1]
Averaging interval	1 hour	Specified in the Caline4 manual [1]
Wind speed	$0.5m/s$ (annual mean wind speed)	Department of Energy and Climate Change [3]
Wind direction	NA	NA for worst case wind angle. Specified in the Caline4 manual [1]
Wind direction standard deviation	NA	NA for worst case wind angle. Specified in the Caline4 manual [1]
Atmospheric stability class	7 (very stable- to simulate worst case scenario)	Specified in the Caline4 manual [1]

Mixing height	$5m$	Specified in the Caline4 manual [1]
Ambient temperature	6 degrees	Met Office annual average for our city [2]
Ambient PM	15.9 micrograms/cubic metre	Background PM_{10} concentration for our city [4]
Link geometry	Excel GIS data export- from PHE GIS shapefiles in ArcGIS	Department for Transport [5]
Link type	At-Grade	Specified in the Caline4 manual [1]
Traffic volume	Excel data import	Department for Transport [5]
Emission factor	0.2g/mile	Emission factor for diesel engine, typical UK HGV fleet [6]

The average wind speed was obtained from the annual mean wind speed provided by the Department of Energy and Climate change[3]. The set of readings were restricted to the cities wards in GIS and the average value was used of 0.5m/s.

Figure A1 shows the road network used to approximate change in particulate matter due to a heavy goods vehicle intervention. R1-R7 (figure A1) are locations at which the air pollution levels are predicted. These locations were chosen to represent urban background levels, being representative of the majority of the urban population. Seven values were arbitrarily chosen at positions close to the A road network (shown in blue). Horizontal and vertical axes are the Easting and Northing coordinates as defined by the AADF dataset respectively[5].

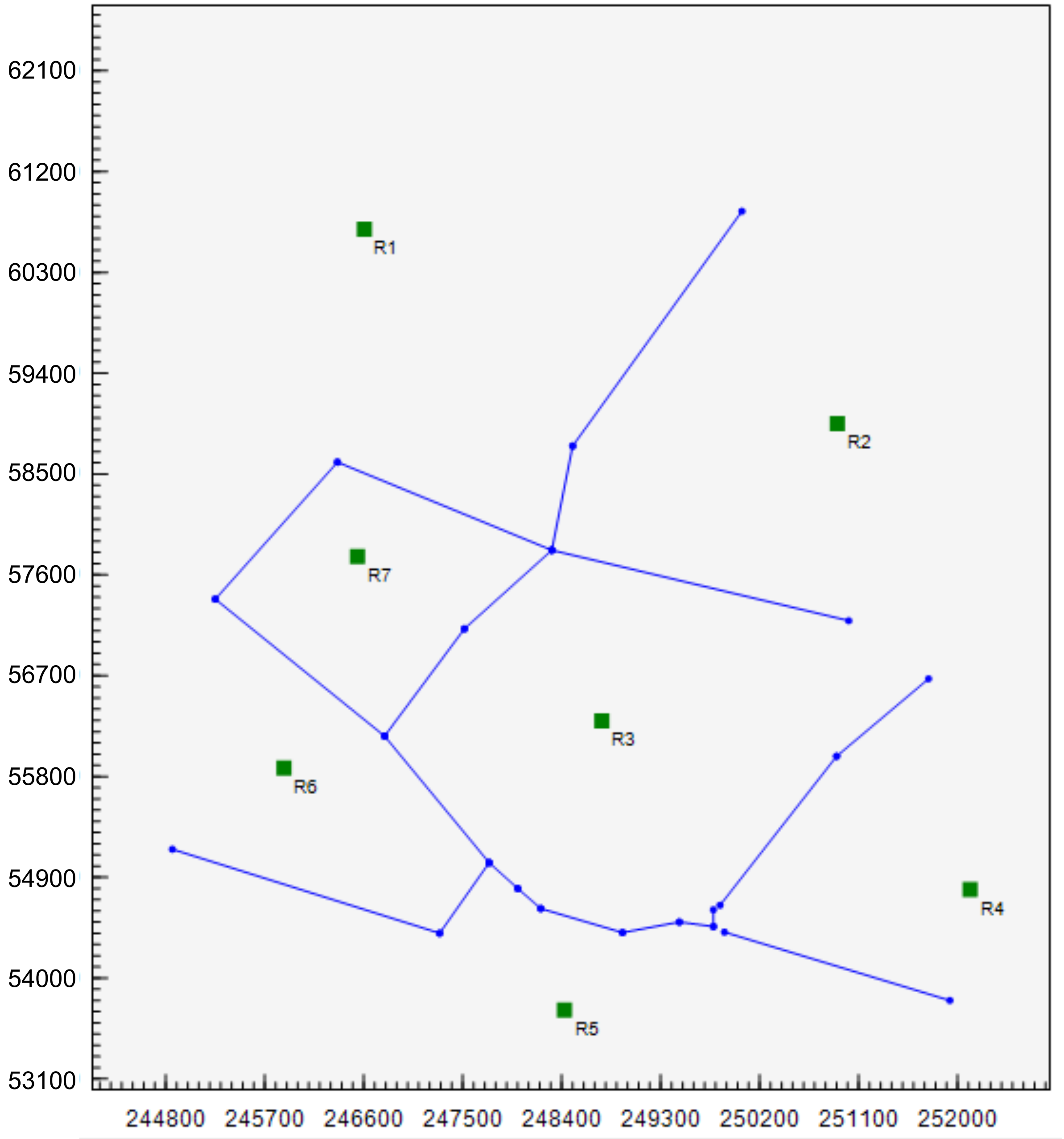

Figure A1. Road network of case study city. Eastings and Northing co-ordinates given.

1. California department of transportation. Caline 4. CL4 v.2.1. http://www.dot.ca.gov/hq/env/air/software/caline4/calinesw.htm
2. Met Office. http://www.metoffice.gov.uk/public/weather/observations
3. Department of Energy and Climate Change. https://restats.decc.gov.uk/cms/annual-mean-wind-speed-map
4. Department for Environment and Rural Affairs. http://uk-air.defra.gov.uk/data/
5. DfT traffic statistics publications. Annual average daily flows; 2012. http://www.dft.gov.uk/traffic-counts/
6. Road Transport Emission Factors. 2011 NAEI RoadtransportEFs_NAEI11_v1.xls; 2013. http://www.dft.gov.uk/publications/road-vehicle-emission-factors-2009
