# Supplementary material for: Decision support for risk prioritisation of environmental health hazards in a UK city
Source: Environ Health. 2016 Mar 8;15(Suppl 1):29. doi: 10.1186/s12940-016-0099-y (PMC4895771; doi:10.1186/s12940-016-0099-y)
Supplement: Additional file 3: — Peer review reports. (PDF 118 kb) [file 12940_2016_99_MOESM3_ESM.pdf]

Reviewer 1

Decision support for risk prioritisation of environmental health hazards in a UK city: Woods et al

This is a useful contribution to the literature on an important and contemporary topic namely decision making across environment and health at a local level in the UK.

Generally the methods and approaches adopted by the authors are well established and consistent with published literature. However commentary on the strengths and weaknesses of the MCDA approach and more discussion of its evolution and application to public health settings is desirable; particularly given MCDA isn't a new methodology and this paper does not adapt it in a radical way.

The modelling seems appropriate, however Eq. 4 (main text) is really a measure of intervention efficacy not effectiveness. This is because the variable E_i is derived from an estimate of an intervention's ability change the exposure levels (Δe_i in Eq. 3, appendix 1), and Eq. 4 does not include any adjustment factor relating to the likely uptake of an intervention e.g:

$$D_i = (E_i \times U_i) \times M_R \times N_i$$

Where U_i is an intervention uptake rate variable which takes a value between 0 and 1, where 1 is complete uptake by the effected population (N_i). This could then perhaps be left as a free parameter to model to derive required uptake rates for an intervention in order to make it the most acceptable one.

While the authors hint at the need for some sensitivity analysis in the discussion, without examining the impact of adjusting the weights and the interactions and varying Δe_i (or U_i) it is difficult to feel confident about the observations.

The text content is clear and concise [a few typos aside] and the tables and figures are easy to read, however it is difficult to find some elements e.g. morbidity component for Radon in the main text and the overall clarity would be enhanced by a table in the body of the main text indicating the values of the variables in Eq. 4 for each intervention for both morbidity and mortality.

The author's discussion and conclusions are generally sound. As discussed earlier It would have been helpful, to include discussion of the current extent of application/use of the MCDA, alternative approaches, and existing practice in the UK.

Also worthy of mention in the discussion is the fit of this work with more strategic approaches including the use of ecological linkage frameworks which more explicitly incorporate the 'distal' determinants of 'health' outcomes and related policy levers across the breadth of local settings.

The funding and relationships are described and there are no obvious issues in relation to the ethical standards of the research.

Summary: a useful contribution to the literature in the UK on an important topic where much work is still needed. Suggest accept subject to the above revisions [discretionary]

Dr David Phillips
Director of Public Health Dorset
Visiting Professor Bournemouth University

Reviewer 2

Review of the Article

Decision support for risk prioritisation of environmental health hazards in a UK city

INTRODUCTION

The article introduces the methodology of a planning and intervention tool to be promoted towards decision makers and stakeholders.

It starts from the need of discipline and policies integration in the field of land planning and it aims at implementing a rational and transparent tool for decision makers.

GENERAL REMARKS

- The objective of the article is highly embraceable and innovative and it is probably urgent! It may be an helpful clue for the need to link scientific evidence and political decisions
- As it is claimed in the article MCDA is aimed at providing clear criteria for decision makers (belonging to public and private sectors) to chose among different options. Hence it would be useful to provide some more comments and suggestions, at least in view of future development, on:
    - Costs-benefits analysis or similar approach which might be useful to be introduced in this field;
    - Economic approach which is surely disputable (eg in terms of ethics), but yearns for objectiveness and transparency;
    - Cost analysis which might be helpful for the private sector, such as insurances.
- Even though it is mentioned in the article, it should be emphasized that the future efforts will be focused in view to the compliance with this approach of decision makers. In other word it should be declared that this work is preliminary and the future endeavours will mainly focus on the friendliness of the product. In fact it will be used by laypersons, and first and foremost it must be easily understood by decision makers who must rely on this approach.

MAJOR REMARKS

1. It's necessary to specify with more details the quality criteria, in particular "robust evidence" and "level of regulation" (page 9). Please note that the definition of the qualitative criteria (reported in the section Results, page 15-16) must be deployed in the section Methods, after the explanation of quantitative criteria). Finally note that at page 20 (in the section Discussion), other 3 criteria are cited which have never been mentioned in section Methods.
2. It's recommended to discuss more deeply the part of environmental hazards and the related interventions as the reader should pay more attention to Table 1.
3. In the section relating to quantitative criteria (page 10), the excess relative risks are referred to all-cause mortality, whilst for some environmental risks presented in the section Results, these excess relative risks are referred to specific-cause mortality. Moreover, in morbidity's formula, the term d_R is defined as incidence rate, whilst in the section Results it is always a prevalence rate.

4. Please pay attention in the section Results as some discussion issues are risen, for instance where some references are mentioned.
5. It would be better to present the results in a more detailed way (for instance by means a table) reporting the elements of the formulas presented in the section Methods (as in Appendix A1).
6. Why morbidity is not considered for Radon? How the 5.3 reduction factor is applied to the baseline level of radon? (see point 5.)

MINOR REMARKS

1. Please use the same abbreviations used in appendix A1 in formulas presented in quantitative criteria section.
2. At the beginning of the section Results, it would be warmly advisable to report, for each hazard, which causes of death and morbidity are considered and which criteria, for example with an explanatory table (see point 5 in major remarks).
3. In the section Results the notation of subscript PPM is not clear.
